# Bac*Dive* in 2019: bacterial phenotypic data for High-throughput biodiversity analysis

**DOI:** 10.1093/nar/gky879

**Published:** 2018-09-26

**Authors:** Lorenz Christian Reimer, Anna Vetcininova, Joaquim Sardà Carbasse, Carola Söhngen, Dorothea Gleim, Christian Ebeling, Jörg Overmann

**Affiliations:** Leibniz Institute DSMZ-German Collection of Microorganisms and Cell Cultures, Braunschweig, Germany

## Abstract

The bacterial metadatabase Bac*Dive* (http://bacdive.dsmz.de) has become a comprehensive resource for structured data on the taxonomy, morphology, physiology, cultivation, isolation and molecular data of prokaryotes. With its current release (7/2018) the database offers information for 63 669 bacterial and archaeal strains including 12 715 type strains. During recent developments of Bac*Dive*, the enrichment of information on existing strains was prioritized. This has resulted in a 146% increase of database content over the past three years. Especially rich datasets were integrated from 4782 manual annotated species descriptions in the *International Journal of Systematic and Evolutionary Microbiology* which yielded standardized phenotypic data for 5468 type strains. Another important improvement of content was achieved through the mobilization of 8977 Analytical Profile Index (API^®^) test results that constitute physiological data for the identification of 5237 strains. Bac*Dive* offers a unique API^®^ data collection with respect to size and diversity. In addition, data on fatty acid profiles and antibiotic susceptibility tests were integrated. A revised graphical user interface and new search tools such as the *API^®^ test finder*, the *TAXplorer*, or the *Microbial Isolation Source Search* significantly improve the user experience.

## INTRODUCTION

Prokaryotes express a wide variety of phenotypic traits with high relevance for research and development. Hotspots for bacterial metadata are the initial species descriptions in primary literature as well as databases maintained by biological resource centers (BRC) (1). Yet, both sources are difficult to access, and searches for particular phenotypic data are often cumbersome. This is of particular relevance, as whole genome sequences are rapidly growing in numbers (to date, 10 957 genomes are found in the NCBI genome database (date accessed 05 July 2018)) and are increasingly used for the prediction and analysis of bacterial phenotypes. Tools like the microbial trait analyzer (2) automatically predict phenotypic attributes from genome sequences and allow a rapid overview over potential phenotypes of the sequenced organisms. Yet, these approaches rely on a framework of homologies to functional sequence information of model organisms, which are often only distantly related to the investigated organism. Therefore, validation of the results with laboratory phenotypic data is indispensable. Moreover, comprehensive and well-structured phenotypic data can be employed to improve the accuracy of the algorithms and thereby enable better predictions and new insights into microbial biodiversity.

Bac*Dive*—the Bacterial Diversity Metadatabase (https://bacdive.dsmz.de) mobilizes, structures and provides metadata across a wide range of bacterial and archaeal strains. The primary sources are internal databases of BRCs (e.g. DSMZ, CIP), unpublished collections of researchers (e.g. Reichenbach collection of Myxobacteria, Wink compendium of Actinobacteria (3)) and species descriptions in primary literature (e.g. *International Journal of Systematic and Evolutionary Microbiology*). By accessing these sources, and curating and combining the relevant data, Bac*Dive* provides comprehensive datasets on the taxonomy, morphology, physiology, cultivation, geographic origin, application and molecular data of prokaryotes. All data are strain-associated and linked to the respective reference.

Since its launch in April 2012 (3), Bac*Dive* has substantially been developed further. The number of data fields has grown, search functionalities were improved and Restful web services were implemented (4). Recent development focused on the amendment of strain datasets with data manually annotated from the primary literature and with data mobilized from internal files of BRCs and private collections (1). The increase in data content and data fields and the accompanying adaptions to the graphical user interface (GUI) and search functionalities are described in the following.

## CONTENT

### Bac*Dive* data statistics

The current Bac*Dive* release (July 2018) covers 63 669 strains distributed over 2637 genera and 13 569 species. 12 715 of the strains included in Bac*Dive* represent type strains of their species; the database thus covers 88% of the 14 395 validly published type strains listed in the Prokaryotic Nomenclature Up-to-Date (PNU) database. Over the past years, enrichment of the existing strain data sets was prioritized over collecting information on new strains. Thus, in comparison to the overall number of strains covered by Bac*Dive* in 2015 (53 978), the increase in new strains amounts to 18% whereas the data content per strain was massively increased in all sections (Table 1). The section *morphology and physiology* (485%), *culture and growth conditions* (391%) and *isolation, sampling and environmental information* (247%) have increased in particular.

**Table 1.** Overview of the increase of data content in Bac*Dive*

Section Name	Selected subjects of the section	Entries 2013*	Entries 2015*	Entries 2018*	Increase in % 2015–2018
Strains total		23 458	53 978	63 669	18%
Name and taxonomic classification	Domain, phylum, class, family, genus, species, subspecies, the full scientific name and type strain status	23 458	103 570	156 229	51%
Morphology and physiology	Size, morphology, utilized substrates, active enzymes, antibiotic resistance, murine types, lysis/ decomposition	11 521	39 087	228 761	485%
Culture and growth conditions	Cultivation media compositions, growth temperatures, pH, salt concentrations	21 605	24 280	119 126	391%
Isolation, sampling and environmental information	Isolation source, geographic location, environmental conditions at sampling time, utilized enrichment media	20 769	22 448	77 883	247%
Application and interaction	Medical, biotechnical or industrial application, risk group classification	14 639	15 680	19 111	22%
Molecular biology	Genotype information e.g. INSDC sequence accession numbers, sequence length, GC-content	14 591	17 363	30 475	76%
Strain availability	Depository history, holding biological ressource center, culture collection identifiers	18 459	66 832	81 373	22%
∑ overall entries		125 042	289 260	712 958	146%

Data are divided into seven thematic sections. Given are the numbers of entries per section in the years 2013, 2015 and 2018 and the percentage increase of entries over the last three years. *) Distinct combination of strain, reference, data entry.

### New types of data

New data types were introduced into Bac*Dive* by integrating data from phenotypic tests, like the Analytical Profile Index (API^®^) tests, fatty acid profiles and antibiotic susceptibility tests. This has resulted in an increase in active data fields from 233 (release September 2015) to 631 (release July 2018).

The API^®^ strip tests are a well-established system of physiological tests for the fast identification of microorganisms. The manufacturer bioMérieux offers 22 test strips, each consisting of up to 50 single tests. We initially mobilized (1) results of 6820 API^®^ tests from internal files of culture collections. Subsequently, API^®^ test results were continuously collected from the ongoing work in the laboratories of culture collections. Currently, Bac*Dive* offers 8977 API^®^ test data, comprising data for 5237 bacterial strains. To our knowledge, this is the worldwide largest collection of API^®^ test results, offering phenotypic information on a large diversity of bacterial strains.

Recently, fatty acid profile and antibiotic susceptibility data were also integrated into Bac*Dive*. Both data types are of great importance for taxonomy and hence a large user group of Bac*Dive*. Initially, 395 fatty acid profiles and 475 antibiotic susceptibility test results were collected. This collection is being continuously amended.

### Literature data

The most comprehensive but at the same time most diverse source for metadata is primary literature. Metadata are scattered all over the different journals. Lacking standardization and unique identifiers, mobilization from literature is challenging. For bacterial and archaeal metadata the *International Journal of Systematic and Evolutionary Microbiology* (IJSEM) is of particular interest, as up to 80% of new species descriptions are published in this journal. Species descriptions in IJSEM are not only rich for phenotypic metadata, but also possess a rather conserved structure. This makes the journal a prime source for the annotation of metadata. We have set up a system for the manual annotation of data for up to 152 different data fields based on species descriptions in IJSEM. These data were transformed using a controlled vocabulary and assigned global identifiers. Initially, data on 523 type strains were collected (1). Since then we proceeded by continuously annotating data from all newly published species descriptions. So far, data on 1387 strains were annotated manually. In 2017, Barberán *et al.* (5) published a data set that comprised annotated data from IJSEM for 5129 strains and for up to 30 data fields. After careful adaptation, data for 4081 strains from this source were incorporated into Bac*Dive*. In the current release, Bac*Dive* offers annotated data from IJSEM species descriptions for a total of 5468 strains (https://bacdive.dsmz.de/advsearch?site=advsearch&searchparams%5B84%5D%5Bsearchterm%5D=IJSEM&advsearch=search).

## USER INTERFACE

The Graphical User Interface (GUI) of Bac*Dive* was completely revised and renewed. The updated GUI offers a responsive design to improve the user experience on mobile devices. Moreover, additional search functionalities and improved export functions have been introduced. The large number of data points added per strain enlarged the *strain detail view* pages, requiring a redesign of these pages and the implementation of additional functionalities. The *strain detail view* offers now a clear and structured access to the extended data provided for each strain.

### Technical concept

The Bac*Dive* database structure consists of a MySQL back end combined with an individual developed PHP front end. The back end is distributed in 94 tables, which ensures a high flexibility in the presentation of the data. This flexibility is needed to address the high variability of bacterial metadata. Therefore, Bac*Dive* offers currently over 600 data fields. Typically, only a fraction of these is used on the individual strain. For a better overview, only filled data fields are displayed. Moreover, the high fractionation of data in the back end allows the easy expansion in new data types (e.g. fatty acid profiles, antibiotic sensitivity tests) that is carried out continuously within the bi-annual updates.

### Strain detail view

**Figure 1.**
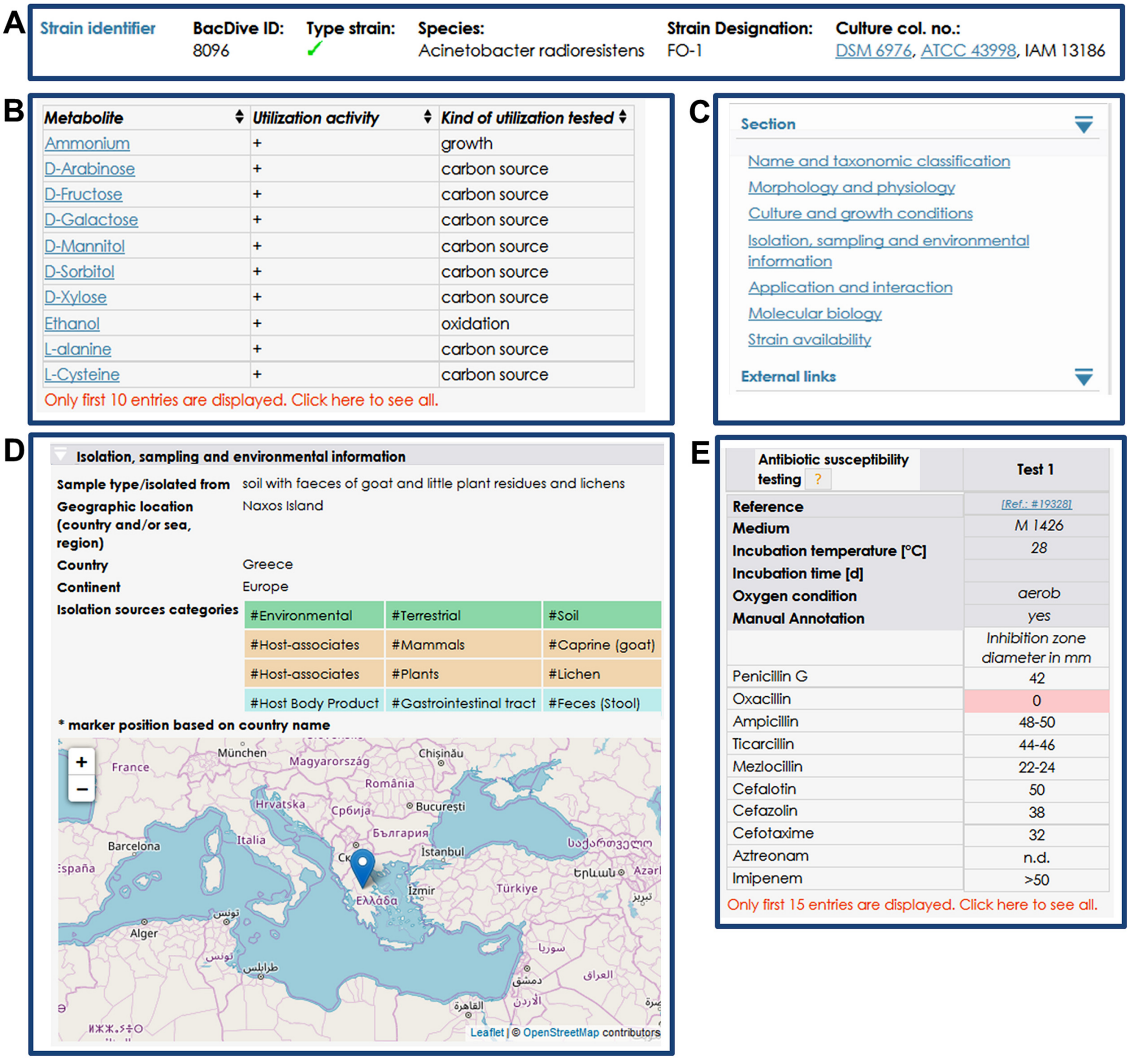
Novel features in the *strain detail view*. (**A**) Infobox comprising main strain identifier. (**B**) Table with metabolite data. (**C**) Navigation bar for a fast switching between thematic sections. (**D**) New isolation source section comprising isolation source category tags enabling systematically search of strains with the new *microbial isolation source search*. (**E**) Table for antibiotic susceptibility test data.

Within the *strain detail view*, an infobox was amended at the top of the page (Figure 1A), providing an overview of the most important strain identifiers including species name, strain designation, and culture collection numbers. For the fast navigation through the single thematic sections, a collapsible navigation bar was integrated at the right side of the *strain detail view* page (Figure 1C). This is followed by the navigation bar that offers links to external web resources and knowledge databases. To improve the overview, the single thematic sections of the *strain detail view* are collapsible. Alternatively, all sections can be collapsed and expanded at once. For a better handling of data fields comprising multiple entries, data tables were integrated that enable sorting of data in the single columns. Long tables (e.g. over 10 entries) are displayed in a shortened preview that can be expanded to display all entries (Figure 1B). Metabolic and enzymatic data within the *strain detail view* were restructured for better query possibilities. For metabolic data, a data entry consists of the metabolite (e.g. glucose), the test result (+/-) and the tested relation (e.g. ‘growth’, ‘builds acid’, ‘electron acceptor’). All metabolites are matched to the Chemical Entities of Biological Interest (ChEBI, (6)) ontology and linked to the according database entry. Where feasible, EC numbers were assigned to enzymes and the entries are linked to the matching entry in the BRaunschweig ENzyme DAtabase (BRENDA, (7)).

In the *strain detail view* the API^®^ test data are presented in tables adapted to the different API^®^ test types. Test results are displayed as ‘+’, ‘+/−’ and ‘−’. The single tests are described in the mouse-over tooltip function and the respective enzyme or metabolite is linked to BRENDA or ChEBI, respectively. Bac*Dive* now offers results for 475 antibiotic susceptibility tests that are integrated into the *strain detail view* of the corresponding strains. The information about the antibiotic susceptibility is displayed in tables that are divided into a metadata header and the results part (Figure 1E). The header comprises data on incubation conditions and the results part is listing the inhibition zone diameters for the respective antibiotic agents. Results with an inhibition zone of a diameter of ‘0’ were classified as resistant. Concentrations of the antibiotics are displayed in the mouse-over function. Data tables for fatty acid profiles are now an additional part of the *strain detail view*. Corresponding to the antibiotic susceptibility tests, the tables of the fatty acid profiles are also divided in a metadata and a result part. Within the metadata, experimental conditions like incubation data, software and instrument information are displayed. In the results part, fatty acids and the percentage share are shown. If available the relative standard deviation (SD) and the estimated chain length (ECL) are displayed. In the section *isolation, sampling and environmental information*, a map was introduced that displays the source of isolation, based on either geographic coordinates (when available) or the country data (Figure 1D). A new feature are isolation source tags that describe the original isolation source entry with terms from a controlled vocabulary and make them systematically searchable (see also *microbial isolation sources search*).

### Search modules

In Bac*Dive*, the *simple search* (https://bacdive.dsmz.de/) is the main tool to quickly find and access information about a specific strain and can be found on the portal main page as well as on the *strain detail view* pages on the upper left corner. The increase in data content made it necessary to extend the existing search functions and to develop new search modules. The *simple search* was extended to cover additional search fields and now permits queries not only of the species name but also of culture collection and INSDC sequence accession numbers. Moreover, strains can be directly accessed by entering the Bac*Dive*-ID.

The *advanced search* (https://bacdive.dsmz.de/advsearch) enables the user to find strains that match a specific phenotype by querying different attributes like culture medium, growth substrates, growth temperature, and GC content. To improve the user experience the *advanced search* query form and the corresponding result sets are now displayed on the same page. Thereby the user can easily adapt the subsequent search pattern based on the previous search results. In 2015, this search was limited to 30 data fields. Meantime, the *advanced search* was extended and now accesses as much as 135 data fields. The search fields within the *advanced search* page are logically structured according to the single thematic sections within Bac*Dive* to improve clarity. All search field sections are collapsible and expandable similar to the *strain detail view* pages. Additional search sections for retrieving specific data were introduced to the *advanced search*. One important amendment is the *antibiotic susceptibility testing* section, that enables queries of the inhibition zone diameters of a total of 36 antibiotic agents. Like all other numeric fields in the *advanced search*, the inhibition zone diameter search allows a filtering for exact matches (‘=’), higher (‘>’) or lower (‘<’) values as well as ranges (‘min-max’). Within the *fatty acid profile* search section, the type of fatty acids and their percentage share, as well as the corresponding experimental data, can be queried. The *reference* search section offers the opportunity to find strains based on the assigned reference (e.g. an IJSEM species description) which can be found via title, authors, journal, year, DOI or directly by entering a Bac*Dive* reference ID. The functions of the *advanced search* are further described within an online tutorial (https://bacdive.dsmz.de/tutorials).

Some data types require specific search options and therefore do not fit into the scope of the *advanced search*. Therefore, separate, powerful and highly adapted search modules were developed that are described below.

The *API^®^ test finder* (https://bacdive.dsmz.de/api-test-finder) is a search tool to retrieve matching test data within the Bac*Dive* API^®^ data collection. In order to start the search, one of the 15 available API^®^ test types has to be chosen first. A test type is a specific combination of physiological tests for the identification of species within a specific group (e.g. *Corynebacteria, Listeria*). Subsequently, a search table specific for each test type is generated including all available data in the Bac*Dive* database for the chosen API^®^ test type. The results for the first 10 entries are displayed. The user may browse page by page or limit the result set by subsequent filtering. In the query form every single test field can now be filtered for positive (‘+’) and negative (‘−’) test results. Ambiguous or variable test fields should be left empty. By filling consecutively all fields in the query form, the list of matching entries and their corresponding strains is narrowed down. Similarly, the results can be further filtered by filling the metadata fields of the query form e.g. with species name or culture collection number. The functions of the API^®^ test finder are further described within the *help* section (reached via ‘?’) and within an online tutorial (https://bacdive.dsmz.de/tutorials).

The *TAXplorer* (https://bacdive.dsmz.de/taxplorer) is an easy to use search tool for browsing the taxonomy of prokaryotic strains in Bac*Dive*. The search allows filtering the strains on all taxonomic levels beginning at the domain level, continuing with phylum, class, order, family, and ending on the genus level. The user is free to start the search on any taxonomic level. From the result list, either the strains can be directly accessed or they can be added to the download selection for data retrieval.

The recently added *microbial isolation sources search* (https://bacdive.dsmz.de/isolation-sources) is a powerful tool to query strains by their isolation sources. The isolation sources are classified according to the controlled vocabulary *Microbial Isolation Source Ontology* (MISO). This ontology is hierarchically ordered into three levels of tags (category 1–3). At the top level the eight major classes #Environmental, #Engineered, #Host, #Host body-site, #Host body-product, #Medical, #Condition and #Climate are listed. By choosing one of these tags, a result list is generated including all matching entries in the Bac*Dive* database. The search can be refined by choosing tags from the second and third level. For example, #Environmental (category 1), #Aquatic (category 2), and #Marine (category 3) can be selected to retrieve all strains that were isolated from marine environments. By submitting such a triplet of tags, the result list gets updated and all strains exhibiting these tags are displayed. Each isolation source is described with up to four triplets. Thereby this tagging system is flexible and allows to describe even complex isolation sources like ‘*soil with faeces of goat and little plant residues and lichens*’ (Figure 1D). Correspondingly, the result list can be further specified by adding more triplets to the filter. Furthermore, the results can be filtered by terms entered in the query form above the result list, e.g. with a distinct species name or a term occurring in the original isolation source description. As in all other search modules of Bac*Dive*, the resulting strains can be either directly accessed or the strains can be added to the download selection for data retrieval.

### Data access

Besides the direct access to data at the *strain detail view* pages, there are several additional ways to retrieve data from Bac*Dive*.

A new function is the PDF archive, which is located in the top right corner of a *strain detail view* page. This archive offers a PDF version for every single *strain detail view* page and all previous versions since the major release in 2017. This allows tracking changes within strain information over time that occur during database updates. Moreover, all PDF files can be found via a stable Digital Object Identifier (DOI). By the introduction of DOIs, Bac*Dive* offers reliable referencing of specific datasets.

Since the first release of Bac*Dive*, the download of data for further analysis is enabled by the *download selection*. Within the *download selection* strains can be collected similar to a shopping cart and then exported in a CSV format. The functionality of the export was enhanced by giving the user the opportunity to filter the exported data fields to own requirements. The exported data can be adapted by selecting from 161 data fields to retrieve individual data sets.

Another option to retrieve data are the web services (https://bacdive.dsmz.de/api/). Two RESTful web services are offered to access data from Bac*Dive* or the PNU database (4).

Moreover, Bac*Dive* serves as data repository for microbial data within the German Federation for the Curation of Biological Data (GFBio (8)), a collaborative effort of German research institutions to build an infrastructure that supports researchers in data management. Thereby, data in Bac*Dive* are connected to the Biological Collection Access Service (BioCase, http://www.biocase.org/), a network of primary biodiversity repositories. Bac*Dive* data are mapped to several data exchange standards e.g. ABCD (https://www.tdwg.org/standards/abcd/), DarwinCore (http://rs.tdwg.org/dwc/) and are accessible via different portals like gfbio.de or GBIF.org. In addition, Bac*Dive* data are available via the BioCASe web service (http://biocase.dsmz.de/).

### Interoperability and interlinking

Currently, great efforts are made to improve the availability (e.g. European Open Science Cloud) and interoperability (FAIR principle (9)) of scientific data. On the data level, interoperability is attained by applying standards and stable identifiers like INSDC sequence accession numbers (10) for sequence data, culture collection numbers for microbial strains or EC numbers for enzymes (http://www.sbcs.qmul.ac.uk/iubmb/). On the database level, reliable links need to be established between databases to enable the user to find associated data quickly. To this end, Bac*Dive* offers linked data to ENA (11), GenBank (12), SILVA (13), BRENDA, GBIF (14), ChEBI and Straininfo.net (15). *Vice versa*, strain-associated links pointing to Bac*Dive* can be found in SILVA to connect the high-quality ribosomal RNA sequence data with curated bacterial metadata. Just recently, links pointing to Bac*Dive* strains were integrated into the NCBI database entries of PubMed, Taxonomy (16) and Nucleotide. These links connect for example 4782 articles with species description in IJSEM (listed in PubMed) with the annotated and structured metadata in Bac*Dive*, or the taxonomy information of 12 084 NCBI taxonomy species with updated information of the PNU database provided by Bac*Dive*, or the 254 947 nucleotide sequences in GenBank with the phenotypic data collected in culture collections available in Bac*Dive*. By providing comprehensive and structured bacterial metadata that are enriched with global identifiers and are available for automated access via web services, the bacterial metadatabase thus enables high-throughput biodiversity analysis.
